# Distance from Healthcare Facilities Is Associated with Increased Morbidity of Acute Infection in Pediatric Patients in Matiari, Pakistan

**DOI:** 10.3390/ijerph182111691

**Published:** 2021-11-07

**Authors:** Elise Corden, Saman Hasan Siddiqui, Yash Sharma, Muhammad Faraz Raghib, William Adorno, Fatima Zulqarnain, Lubaina Ehsan, Aman Shrivastava, Sheraz Ahmed, Fayaz Umrani, Najeeb Rahman, Rafey Ali, Najeeha T. Iqbal, Sean R. Moore, Syed Asad Ali, Sana Syed

**Affiliations:** 1 Department of Pediatrics, School of Medicine, University of Virginia, Charlottesville, VA 22903, USA; ecc8z@virginia.edu (E.C.); ys5hd@hscmail.mcc.virginia.edu (Y.S.); farazraghib7@gmail.com (M.F.R.); fatimaz@virginia.edu (F.Z.); lubaina.ehsan@gmail.com (L.E.); as3ek@virginia.edu (A.S.); srm5u@hscmail.mcc.virginia.edu (S.R.M.); 2 Department of Paediatrics and Child Health, Aga Khan University, Karachi 74800, Pakistan; saman.siddiqui@aku.edu (S.H.S.); sheraz.ahmed@aku.edu (S.A.); fayaz.umrani@aku.edu (F.U.); najeeb.rahman@aku.edu (N.R.); rafey.ali@aku.edu (R.A.); najeeha.iqbal@aku.edu (N.T.I.); 3 Department of Engineering Systems and Environment, University of Virginia, Charlottesville, VA 22903, USA; wa3mr@virginia.edu; 4 Department of Public Health Sciences, University of Virginia, Charlottesville, VA 22903, USA; 5 School of Data Science, University of Virginia, Charlottesville, VA 22903, USA

**Keywords:** nutrition, pediatrics, geographic information systems, acute respiratory infections, diarrhea, growth

## Abstract

The relationship between environmental factors and child health is not well understood in rural Pakistan. This study characterized the environmental factors related to the morbidity of acute respiratory infections (ARIs), diarrhea, and growth using geographical information systems (GIS) technology. Anthropometric, address and disease prevalence data were collected through the SEEM (Study of Environmental Enteropathy and Malnutrition) study in Matiari, Pakistan. Publicly available map data were used to compile coordinates of healthcare facilities. A Pearson correlation coefficient (*r*) was used to calculate the correlation between distance from healthcare facilities and participant growth and morbidity. Other continuous variables influencing these outcomes were analyzed using a random forest regression model. In this study of 416 children, we found that participants living closer to secondary hospitals had a lower prevalence of ARI (*r* = 0.154, *p* < 0.010) and diarrhea (r = 0.228, *p* < 0.001) as well as participants living closer to Maternal Health Centers (MHCs): ARI (*r* = 0.185, *p* < 0.002) and diarrhea (*r* = 0.223, *p* < 0.001) compared to those living near primary facilities. Our random forest model showed that distance has high variable importance in the context of disease prevalence. Our results indicated that participants closer to more basic healthcare facilities reported a higher prevalence of both diarrhea and ARI than those near more urban facilities, highlighting potential public policy gaps in ameliorating rural health.

## 1. Introduction

Children in rural areas of countries often lack ready access to adequate healthcare. In Pakistan, a country that spends only 3.4% of its total gross domestic product (GDP) on healthcare, health-related resources are often described to be insufficiently funded or poorly allocated [1]. Healthcare in Pakistan is delivered through a three-tiered system. The first tier, primary care centers, includes Basic Health Units (BHUs) and Rural Health Centers (RHCs). These facilities focus on preventive, curative, and referral services. RHCs also maintain a small number of inpatient beds. The second tier, secondary care centers, provides both ambulatory and inpatient care. Examples include Tehsil Headquarter Hospitals and District Headquarter Hospitals. The third tier, teaching hospitals, includes tertiary care centers that provide specialized inpatient care and receive referrals from other healthcare centers [1].

One reason for the stark health disparities in Pakistan is that many healthcare facilities are located in more densely populated urban areas. Thus, rural populations have disproportionately low access to healthcare. For example, Matiari, a rural town in Pakistan’s Sindh province, has been burdened by chronic poverty. In addition, the roadway infrastructure in Matiari is severely underdeveloped, consisting of only 178 km (48 mi) of quality roads within the district’s 141,000 hectares (544 sq mi). The closest large city, Hyderabad, is thirty kilometers away along the only national highway in the region [2]. This lack of road infrastructure creates a significant barrier to access to healthcare, and so in Matiari and other remote areas, these transportation-related difficulties and a high disease prevalence combine to affect the health of these rural populations adversely.

This study has investigated the relationship between geospatial factors and the morbidity of acute infections in the pediatric population, specifically acute respiratory infections (ARIs) and diarrhea. The adverse impact of distance in healthcare is well documented. Distance from healthcare has been associated with higher neonatal mortality [3]. In South Africa, a complex relationship between race and distance to healthcare facilities has shown that respondents with poorer income status are more likely to live up to 0.75 km further from the nearest healthcare facility than those in higher income brackets [4]. In Malawi, distance from healthcare facilities was associated with decreased malaria prevention practices, such as possession of a mosquito net. Families living further from healthcare facilities were less likely to possess a mosquito net [5].

Additionally, a lack of infrastructure exposes patients traveling to healthcare facilities to inclement weather and other hazardous conditions, which then exacerbates the inaccessibility of basic services. This, in turn, impacts acute care and long-term health outcomes, such as vaccination rates [6]. Thus, the further one is from a healthcare facility, the less likely they are to use the facility [7]. Therefore, we hypothesize that a greater travel distance between pediatric patients and healthcare facilities is associated with higher morbidity due to acute infection.

We have used geographical information systems (GIS) technology to explore the relationship between health and distance to healthcare. GIS technology comprises geospatial analytical platforms that are “computer-based system(s) used for collecting, editing, visualizing, and analyzing spatially-referenced data [8].” Enabling the visualization of disease trends and disease clustering concerning geographic proximity, GIS provides valuable information on the relationship between disease and geography, clarifying the interplay of so-called “socioecological exposure” and illness [9]. In recognizing the multi-faceted nature of healthcare accessibility, we have employed random forest regression kriging analysis to study the complex interplay of other factors that determine child health. This research aims to test the hypothesis that increased distance to health services is associated with an increased risk for child health outcomes such as ARIs, diarrhea, and growth. We also hypothesize that distance to more complex, tertiary healthcare centers is associated with a decreased burden of disease. Finally, we aim to explore the effects of other socio-demographic variables collected in our study on the childhood morbidity of acute infections.

## 2. Materials and Methods

### 2.1. Study Participant Enrollment

Data were collected via the Study of Environmental Enteropathy and Malnutrition (SEEM), a prospective inception cohort study investigating environmental enteropathy in Matiari, Pakistan. Study participants were recruited through community-based surveillance and monitoring of the population. The study enrolled 416 children under the age of 2 in Matiari between 2016 and 2019. Detailed recruitment methods and inclusion and exclusion criteria are detailed elsewhere in the study by Iqbal et al. [10]. Anthropometric data and disease prevalence data were collected by community health workers who visited the children’s homes. Subjects were followed for 24 months or until a study participant reached two years of age. Weekly visits included a survey documenting daily symptoms of diarrhea or ARI.

### 2.2. Geographical Data Collection of Healthcare Facilities and Participant Residence

A comprehensive list of healthcare facilities in Matiari was compiled by searching historical records and using up-to-date satellite imaging through accessing publicly available map data. We identified a total of 21 healthcare facilities, including 7 Basic Health Units (BHUs); 6 Dispensaries; 1 District Headquarter Hospital; 1 Taluka Hospital; 3 Rural Health Centers (RHCs); and 3 Maternal Health Centers (MHCs). The District Headquarter Hospital and Taluka Hospital were grouped as secondary care hospitals. Spatial coordinates of each healthcare facility were acquired using Google Earth satellite imaging and were recorded as latitude and longitude. In addition, the spatial coordinates of each subject’s home were collected upon enrollment in the study. The distance of each subject to the nearest healthcare facility was computed in kilometers (km).

### 2.3. Defining Clinical Outcomes: ARI Events, Diarrhea Events, and Growth Parameters

The threshold for diarrhea or ARI events was established when a child showed signs and symptoms for a minimum of 2 days, followed by a 7-day symptom-free interval. A child was determined to have had a “diarrhea day” when they excreted three or more loose or liquid stools in one day. An ARI day occurred when a subject reported cough or shortness of breath. The prevalence of diarrhea and ARI events was recorded as the number of sick days/observed days multiplied by 365 days. This value was used to represent morbidity. At monthly visits, the community health workers also obtained weight and length measurements, which were used to contrast with the nutritional status and growth of the child. These measurements were used to calculate the height-for-age Z-score (HAZ), weight-for-age Z-score (WAZ), and weight-for-height Z-score (WHZ). The nutritional status of the children was also closely monitored. We want to note that nutritional intervention in the form of education was provided to the parents when the child was at 6 months of age. At 9 months of age, if a child was more than 2 standard deviations below the WHZ, they received nutritional intervention in the form of a supplement at 10 and 11 months. If the study participants were less than 2 standard deviations below the WHZ, they continued with weekly and monthly follow-ups by the community health workers [10].

### 2.4. Calculation of Anthropometric Data and Correlation Analysis

For our correlation analysis, changes in growth, or deltas, were calculated for the anthropometric variables, measuring the difference from 0–6 months to 24 months of age. All continuous variables, including the prevalence of diarrhea, the prevalence of ARI, delta weight, delta HAZ, delta WAZ, and delta WHZ, were expressed in mean (±standard deviation; S.D.).

The Pearson correlation coefficient (*r*) was used to calculate the correlation between distance from healthcare facilities and study participant growth and the prevalence of ARI and diarrhea. Significance was tested with a Student’s *t*-test on the calculated Pearson’s correlation coefficient. A *p*-value of less than 0.05 was considered statistically significant.

### 2.5. Random Forest Regression Modeling of Other Continuous Variables

**Figure 1:**
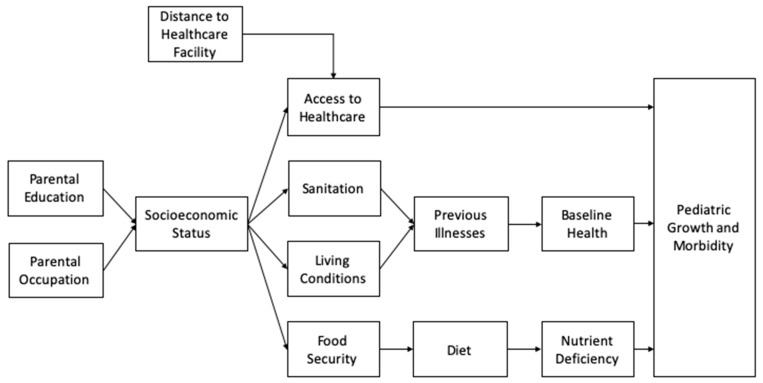
Causal pathways depicting the relationships between various parameters and pediatric growth and morbidity.

We recognized that factors other than geographic distance from hospitals covary with growth and morbidity in pediatric patients. Data show that children in lower socioeconomic groups have a greater incidence of disease morbidity worldwide [11,12]. Therefore, we increased the scope of our project to investigate other factors influencing the occurrence of acute infections in pediatric patients. To adopt a holistic approach in our investigation of factors contributing to disease burden, we have analyzed the relative contribution of other environmental variables such as household caste and parental demographics to determine their relative impact on the outcomes we established for this project. First, a conceptual causal pathway was created to depict the multi-faceted relationship of the various determinants of pediatric growth and morbidity (Figure 1).

Next, random forest regression kriging analysis was used to study the association between the continuous environmental variables and identified growth or morbidity parameters, including distance to healthcare facilities. Random forest analysis is a commonly used machine learning model that uses many decision trees working together “as an ensemble.” Random forest regression kriging, specifically, has been adopted in a number of studies as a way to account for spatial autocorrelation in random forest modeling [13,14]. The regression model was trained to predict correlated parameters using distance, growth, and other clinically relevant variables such as parental caste, number of household members, and maternal age. Feature importances were extracted from the trained random forest model to rank features with the intent of further exploring these features as potential confounders in the assessment of distance and disease prevalence. To maximize the training dataset for training and testing the model performance, an 80–20 split of data was used, respectively. Our random forest regression model was created using sklearn’s Random Forest Regressor packages, and the PyKrige package (Copyright 2017–2021, PyKrige developers. Revision f5b39204) was used for regression kriging and implemented in Python [15]. In random forest analysis, the number of trees was set to 100, and the remaining parameters were kept as default in sklearn’s implementation.

## 3. Results

### 3.1. Descriptive Summary of Data Collection

**Figure 2:**
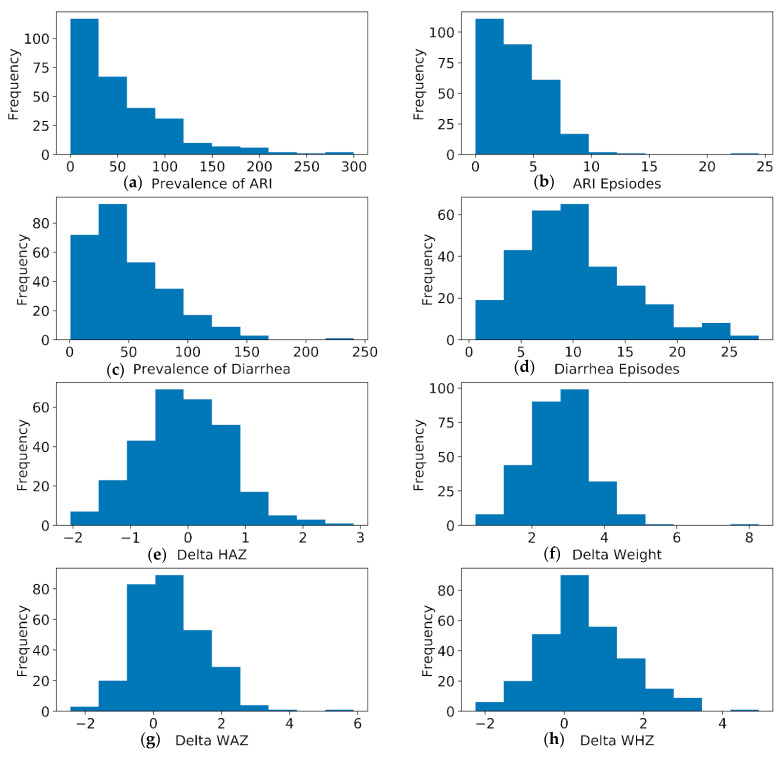
Histogram explaining the distribution of anthropometric variables and prevalence variables. (**a**) Prevalence of acute respiratory infections (ARIs) (**b**) number of ARI episodes (**c**) Prevalence of diarrhea; (**d**) Number of diarrhea episodes (**e**) change (delta) height for age (HAZ) score (**f**) Change (delta) in weight (**g**) change (delta) weight for age (WAZ) score (**h**) Change (delta) in weight (**g**) change (delta) weight for height (WHZ) score.

Our study included 416 children (61% male) with a mean age at enrollment of 4.2 mo (±1.0 mo). The mean distance from healthcare centers was 2.3 km (±1.1 km), and the mean weight difference from 0–6 till 24 months was 3 kg (±0.9 kg). Additional background demographic data are presented in Table 1. The prevalence of diarrhea and ARI among the 297 participants was 50% (±34.1) and 54% (±54), respectively. A histogram explaining the distribution of anthropometric variables and prevalence variables is provided in Figure 2.

### 3.2. Prevalence of ARI and Diarrhea Correlates with Study Participant Distance from Healthcare Facilities

**Figure 3:**
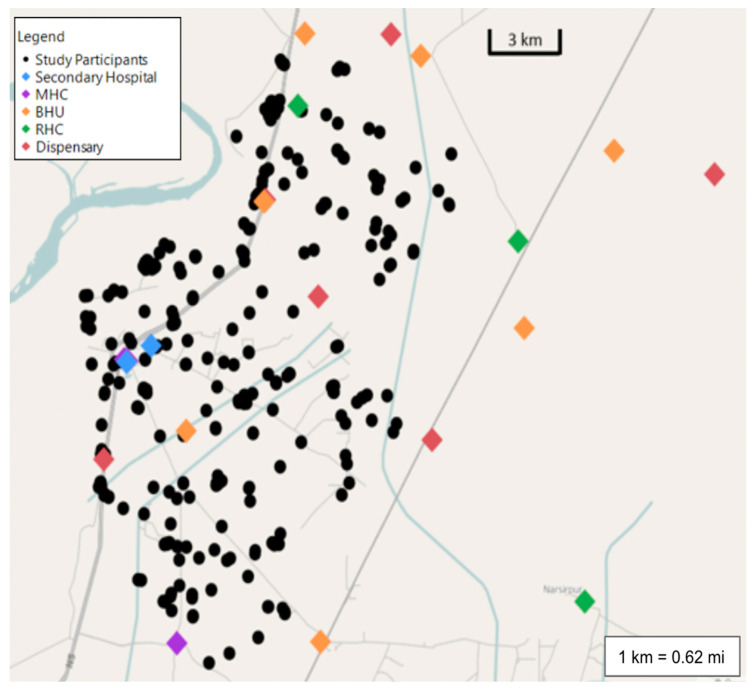
Map of healthcare facilities and study participants in Matiari, Pakistan. MHC: Maternal Health Center; BHU: Basic Health Unit; RHC: Rural Health Center. Data were collected via the Study of Environmental Enteropathy and Malnutrition (SEEM), a prospective inception cohort study that investigated environmental enteropathy in Matiari, Pakistan, maps.google.com, and reliefweb.int (accessed on 24 August 2020).

Table 2 reports the relationship between growth parameters and the prevalence of acute infections and the correlation between morbidity and distance from healthcare facilities. Our results indicate a positive relationship between ARI and distance, suggesting that participants located closer to secondary hospitals have a lower prevalence of both ARI (*r* = 0.154, *p* < 0.010) and diarrhea (*r* = 0.228, *p* < 0.001). Our results revealed a similar relationship between disease burden and proximity to MHCs. Participants living closer to MHCs had a statistically significant decrease in the prevalence of ARIs (*r* = 0.185, *p* < 0.002) and diarrhea (*r* = 0.223, *p* < 0.001). Conversely, subjects that lived near BHUs and RHCs demonstrated a higher burden of disease. The prevalence of ARI was increased in patients living in closer proximity to BHUs (*r* = −0.126, *p* = 0.034) and RHCs (*r* = −0.212, *p* < 0.001). Additionally, proximity to RHCs was also associated with an increased prevalence of diarrhea (*r* = −0.258, *p* < 0.001). There was no statistically significant relationship to report between the distance from any type of healthcare facility and the delta weight, delta HAZ, delta WAZ, or delta WHZ scores (Table 2). Proximity to dispensaries also did not play a statistically significant role in daily morbidity or growth. A map of participant proximity to various types of healthcare facilities shown in Figure 3 and detailed coordinated for each healthcare facility can be found in Supplementary Table S1.

### 3.3. Distance Has the Highest Variable Importance in ARI and Diarrhea Outcomes

**Figure 4:**
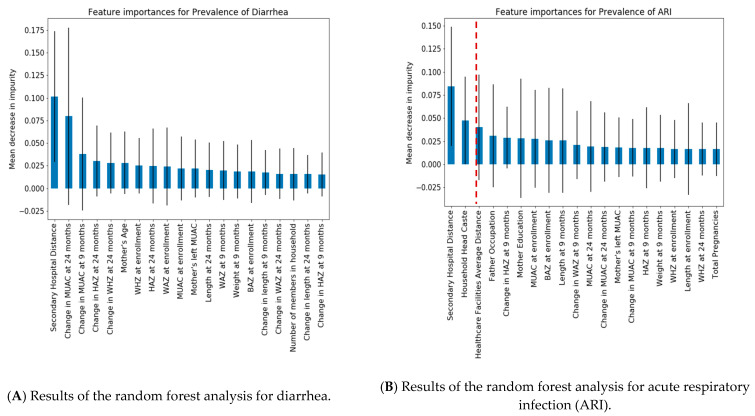
Feature importance graphs for ARI (**A**) and diarrhea (**B**). Note: The mean decrease in impurity is a feature importance metric describing the improvement in predictability observed due to each variable. Based on the importance score and ranking, distance-based features were most important in both ARI and diarrhea, followed by the change in the child’s MUAC for diarrhea. All the other features (after the red dashed line) showed high standard deviations, which in some instances was a negative value and, in most instances, crossed zero. These high standard deviations, combined with low feature importance, suggest that these features had limited to no contribution to the predictability of the prevalence of ARI or diarrhea. MUAC = Mid upper arm circumference; HAZ = Height-for-Age Z-score; BAZ = Body Mass Index for Age Z-score; WHZ = Weight-for-Height Z-score; WAZ = Weight-for-Age Z-score.

In our random forest analysis of variables deemed contributory to an increased prevalence of diarrhea and ARI or growth parameters, we found that distance was the feature that was found to have the highest variable importance in relation to the prevalence of both ARIs and diarrhea. It was also observed that the change in mean upper arm circumference (MUAC) at 24 months was found to be a feature of importance in relation to diarrhea, but not in ARI. Other features, such as household caste, father’s occupation, and the WHZ and HAZ scores at various age points, did not appear as important on our model (Figure 2). The mean decrease in impurity is a feature importance metric describing the improvement in predictability observed due to each variable. Based on the importance score and ranking, distance-based features were the most important for both ARI and diarrhea. All the other features (Figure 4, after the red dashed line) showed high standard deviations, which in some instances was a negative value and, in most instances, crossed zero. These high standard deviations, combined with low feature importance, suggest that these features had limited to no contribution to the predictability of the prevalence of ARI or diarrhea.

## 4. Discussion

In this study, we present a framework for the use of geospatial data to explore the relationship between a patient’s geographic proximity to healthcare facilities and the prevalence of ARI and diarrhea as well as growth. We have also presented a framework to minimize the confounding effects of a myriad of other variables, such as maternal and paternal sociodemographic characteristics, by using a random forest regression model to show that distance from healthcare facilities has high relative importance in increased morbidity from ARIs and diarrhea.

Our results indicated that the distance to various tiers of healthcare facilities was associated with different disease-related outcomes. Study participants located closer to RHCs and BHUs reported a higher prevalence of both diarrhea and ARI as compared to secondary and tertiary care facilities. A review of the map of healthcare facilities reveals that RHCs and BHUs are more likely to be found in more rural areas of Matiari, providing a socio-ecologic explanation for the increased disease prevalence in these areas (Figure 3). Thus, while proximity to healthcare facilities may have some role in child health, our data suggest that living in more rural areas may also negatively impact childhood morbidity. Conversely, children living closer to secondary hospitals and MHCs had a lower prevalence of diarrhea and ARI. Notably, the secondary hospitals and MHCs are located in more urban areas and along main roads, thus making them more accessible to subjects (Figure 3). Our random forest analyses provide further evidence that the distance between subjects and healthcare facilities is related and has an impact on pediatric morbidity (Figure 2).

Although 70% of Pakistan’s population lives in rural regions, the vast majority of healthcare facilities and 85% of doctors are located in more populated areas [10]. This discrepancy places a fiscal and infrastructure burden on rural provinces such as Sindh. There are three doctors for every 10,000 residents in the Sindh province, while Pakistan has seven doctors for every 10,000 residents [16]. The urban versus rural healthcare imbalance can be further demonstrated by the fact that 23% of Sindh’s healthcare facilities, which hold 40% of the beds, are located in the six districts of urban Karachi, the largest city in Pakistan [1]. The scarcity of healthcare facilities in rural Pakistan often necessitates extensive travel for patients to seek care. Physical distance has been recognized as a significant barrier to accessing healthcare, as previous studies have concluded that distance has the potential to negatively impact the overall health outcomes of patients [17]. While extended travel is challenging for any ill patient, it is challenging for patients who live in remote areas. These areas may lack adequate road systems and have a challenging topography [18]. The road network infrastructure in Matiari is severely underdeveloped, and most individuals travel on foot or by rickshaw, making travel with young children difficult, dangerous, and expensive [19]. Puett et al. noted that Sindh weather conditions and seasonality could pose significant challenges, and patients traveling on foot are at risk of exposure to the elements. They reported that frequent flooding during the rainy season and higher temperatures in the summer months could dissuade families from traveling to seek care [18]. On average, the subjects enrolled in our study lived 2.25 km away from the closest healthcare facility, making travel on foot even more difficult. In addition, once families transport the child, they arrive at healthcare facilities that serve a larger geographic area consisting of a sizeable patient population. Therefore, parents who have overcome the burden of travel are likely to have long wait times. This places an additional burden on patients and family members, as they must sacrifice a significant amount of time to travel to and wait to receive care [19].

Our random forest analyses support the hypothesis that the distance to healthcare facilities is associated with childhood morbidity. Furthermore, using our random forest analysis, we aimed to clarify the complex interplay between various socioeconomic factors and the health of this population of pediatric patients. The relationship between socioeconomic status and health has been extensively described in the literature, and it has been established that lower status has a negative impact on the physical health of children [11,12,19]. The main factors contributing to socioeconomic status include household income, education, and parental occupation, and research has shown that parental socioeconomic status is intrinsically connected to their children’s outcomes [20]. Furthermore, a global view reveals significant gaps in childhood mortality between high and low–middle income countries, a trend that extends to inter-country populations with wealth disparity. This disparity has been attributed to lower disease resistance in impoverished children due to poor nutrition, hazardous living conditions, and reduced access to healthcare [21]. Unfortunately, the relationship between health and socioeconomic status represents a negative feedback loop, where frequent or severe illnesses may harm one’s social position [12]. However, in our random forest analysis, the relative importance of these features was low, and their standard deviations were high, indicating that based on our data, these social features had limited association with the prevalence of diarrhea and ARI (Figure 3).

In Pakistan, urban facilities, such as District Headquarters and Tehsil Hospitals, employ numerous staff members and are frequently visited. In contrast, facilities in remote regions are more susceptible to severe damage or unreported abandonment due to lower utilization and a lack of staff. Even within staffed and functioning facilities, the services and supplies required to provide comprehensive care are often extremely limited [22]. Inpatient facilities, usually with limited beds, are often burdened with overcrowding, while outpatient clinics consistently have long wait times [22]. These shortcomings impact pediatric patients especially hard as children are among the most vulnerable patient populations. Given that only 42% of facilities offer pediatric services, pediatric growth monitoring is not widely available. On average, 52% of facilities can assess and treat ARI and diarrhea in children under 5, and only 36% can assess and manage childhood nutrition [22,23]. We have provided data that suggest that the distance to healthcare facilities may be another factor adding to the burden of child health in Pakistan.

To our knowledge, this is the first study to examine the relationship between proximity to healthcare centers and childhood morbidity and growth in the Sindh region of Pakistan. We have outlined a framework for the use of GIS technology to explore these variables in other similar populations. This work highlights the need for future efforts to improve access to healthcare facilities for rural patient populations. Additionally, this study prompted the establishment of an up-to-date and complete list of healthcare facilities in Matiari, Pakistan, which will aid in future research efforts in this area. However, our study was also faced with several limitations. Although the original data were collected via SEEM, a prospective inception cohort study, our data analysis was performed retrospectively. Thus, we would like to have collected data points of interest if real-time analysis had taken place. There were no data collected regarding in which healthcare centers the study participants received their care. Therefore, we should note that our analysis assumes that subjects sought care at the government-funded healthcare facilities closest to their place of residence and not private healthcare institutions. In addition, straight-line distance computed using latitude and longitude was used when calculating the distance from healthcare facilities, with the assumption that, in rural areas, there exist multiple shortcuts and off-road routes that are not directly reflected in the road distance approach. Further, since we are dealing with a rural population, there is a high probability many of the participants would have taken a walking route instead of a road. While this assumption is likely given that Matiari is a rural area and most transportation takes place by foot, it cannot be confirmed. Additionally, it was assumed that subjects did not move residence for the duration of the study. Furthermore, healthcare facilities were grouped based on their type, which assumes that the care provided at each type of healthcare facility in one group is roughly equal. Another critical component of rural healthcare infrastructure in Pakistan is community-based lady health workers (LHWs). LHWs provide limited preventive and curative maternal and child health services, including childhood immunizations and essential treatment of diarrhea and ARI. The eligibility criteria for the recruitment of LHWs are minimum 8th grade followed by a 15-month training program. In Matiari, more than 500 LHWs work to cover 60% of the area, again covering more populated areas. We have not included the density of LHWs and the populations they serve in our model [24]. Other potential confounding factors to be addressed in future studies include cultural and social factors such as healthcare literacy, willingness to seek care, and beliefs in alternative medicine.

## 5. Conclusions

Many factors contribute to the growth and health of pediatric patients, including proximity and access to quality healthcare and timely nutritional and medical interventions. Increased distance from such facilities was found to contribute to the increased morbidity of ARI and diarrhea in children in Matiari, Pakistan. Given the imbalance of urban and rural healthcare facilities in the Pakistani healthcare system, we conclude that it is exceedingly difficult for patients living in remote areas to access quality healthcare. This study highlights the need for comprehensive healthcare reform in developing countries, specifically in remote areas with a high burden of disease morbidity. Further exploration of variables associated with poor health outcomes must be carried out to improve our understanding of the pitfalls of accessing quality healthcare and the future directions for public health experts, policymakers, and stakeholders to improve health in these rural populations.

## Figures and Tables

**Table 1:** Sociodemographic and presentation characteristics of subjects enrolled in SEEM (Study of Environmental Enteropathy and Malnutrition) study between 2016 and 2019 in Matiari, Pakistan (*n* = 416, 61% male).

Variable	Mean (±SD)
**Age at Enrollment (months, (mo))**	**4.2 (±1.0)**
HAZ at Enrollment (mo)	−2.3 (±1.4)
WAZ at Enrollment (mo)	−3.6 (±1.4)
WHZ at Enrollment (mo)	−2.6 (±0.7)
Weight Difference from 0–6 to 24 months (kg)	3.0 (±0.9)
Prevalence of ARI per year *	54.2 (±53.3)
ARI Episodes **	5.0 (±3.6)
Prevalence of Diarrhea per year ***	50.0 (±34.3)
Diarrhea Episodes ****	14.8 (±17.7)
Distance from Healthcare Facility	2.3 (±1.1) km 1.4 (±0.7) mi
Distance from Secondary Hospital	6.5 (±3.5) km 4.0 (±2.2) mi
Distance from Maternal Health Center	6.3 (±3.6) km 3.9 (±2.2) mi
Distance from Basic Health Unit	3.6 (±1.4) km 2.2 (±0.9) mi
Distance from Rural Health Center	9.0 (±4.8) km 5.6 (±3.0) mi
Distance from Dispensary	4.0 (±1.8) km 2.5 (±1.1) mi

HAZ = height-for-age Z score. WAZ = weight-for-age Z score. WHZ = weight-for-height Z score. ARI = Acute Respiratory Infection. ARI days = Subject reporting cough and/or shortness of breath. * Prevalence of ARI = (number of ARI days/observed days) × 365. ** ARI episode = ARI for minimum of 2 days with signs followed by a sign-free interval of 7 days. Diarrhea day = Subject excreting three or more loose or liquid stools in one day. *** Prevalence of Diarrhea = (Number of Diarrhea days/observed days) × 365. **** Diarrhea Episode = New episode is defined as not meeting the diarrheal definition for at least 2 days. km = kilometers, mi = miles. One kilometer = 0.623 miles.

**Table 2:** Correlation of growth parameters and prevalence of acute infections with distance from healthcare facilities.

Variable	Secondary Hospital		Maternal Health Center		Basic Health Unit		Rural Health Center		Dispensary	
	Correlation Coefficient	*p*-Value	Correlation Coefficient	*p*-Value	Correlation Coefficient	*p*-Value	Correlation Coefficient	*p*-Value	Correlation Coefficient	*p*-Value
**ARI Episodes**	0.057	0.334	0.067	0.258	−0.081	0.176	−0.154	0.009	0.022	0.711
**Prevalence of ARI**	0.154	0.009	0.185	0.002	−0.126	0.034	−0.212	<0.001	−0.044	0.466
**Diarrhea** **Episodes**	0.151	0.010	0.163	0.006	−0.079	0.183	−0.260	<0.001	−0.057	0.335
**Prevalence** **of Diarrhea**	0.228	<0.001	0.223	<0.001	−0.044	0.459	−0.212	<0.001	−0.005	0.940
**Delta Weight**	−0.039	0.515	−0.049	0.416	−0.108	0.070	0.066	0.271	−0.026	0.662
**Delta HAZ**	0.067	0.260	−0.014	0.816	−0.104	0.080	0.099	0.097	−0.009	0.885
**Delta WAZ**	0.008	0.893	−0.021	0.723	−0.087	0.144	0.074	0.215	−0.046	0.442
**Delta WHZ**	−0.044	0.464	−0.024	0.687	−0.041	0.497	0.030	0.617	−0.042	0.477

Results of Student’s *t*-test on Pearson’s correlation coefficient. *p* < 0.05 considered significant. ARI = Acute Respiratory Infection. ARI day = Subject reporting cough and/or shortness of breath. Prevalence of ARI = (number of ARI days/observed days) × 365. Diarrhea day = Subject excreting three or more loose or liquid stools in one day. Prevalence of Diarrhea = (number of diarrhea days/observed days) × 365. HAZ = height-for-age Z score; WAZ = weight-for-age Z score; WHZ = weight-for-height Z score. Delta indicates the change over time from subject enrollment to study completion at 24 months of age.

## Data Availability

This study used data collected as part of the Study of Environmental Enteropathy and Malnutrition (SEEM).

## Supplementary Materials

The following are available online at https://www.mdpi.com/article/10.3390/ijerph182111691/s1. Supplementary Table S1: Comprehensive list of healthcare facilities in Matiari, Pakistan.
