# Supplementary material for: Distance from Healthcare Facilities Is Associated with Increased Morbidity of Acute Infection in Pediatric Patients in Matiari, Pakistan
Source: Int J Environ Res Public Health. 2021 Nov 7;18(21):11691. doi: 10.3390/ijerph182111691 (PMC8583418; doi:10.3390/ijerph182111691)
Supplement: Supplementary file 1 [file ijerph-18-11691-s001.zip › ijerph-1429432-supplementary.pdf]

Supplementary Table S1. Comprehensive list of healthcare facilities in Matiari, Pakistan

Union Council	Health Facility Name	Health Facility Type	Latitude	Longitude
Bao Khan Pathan	BHU Bao Khan Pathan	BHU	25.607036	68.598257
Faqir Nooh Hothiani	BHU Faqir Nooh Hothiani	BHU	25.705407	68.513972
Oderolal Village	BHU Tahir Hinngoro	BHU	25.666246	68.632816
Oderolal Village	BHU Oderolal Village	BHU	25.697929	68.558528
Sekhat	BHU Sekhat	BHU	25.649272	68.498243
Shah Alam Shahwari	BHU Laloo Mangwano	BHU	25.572692	68.468336
Tajpur	BHU Allah Dinosand	BHU	25.50227	68.519977
Bao Khan Pathan	G.D. Shahmeer Jiwari	Dispensary	25.61755	68.519116
Bao Khan Pathan	DCD Palijani	Dispensary	25.569672	68.562855
Faqir Nooh Hothiani	G.D. Sher Muhammad Thorha	Dispensary	25.705117	68.54707
Oderolal VIllage	G.D. Dhaloo Khaskeli	Dispensary	25.658382	68.671428
Sekhat	G.D. Juma Shaok	Dispensary	25.64985	68.498859
Shah Alam Shah	G.D. Soomro Chad	Dispensary	25.563194	68.436686
Matiari	Matiari Health Services	Private Hospital	25.601161	68.454878
Matiari	Taluka Hospital Matiari	Taluka Hospital	25.595665	68.445469
Matiari	Taluka Hospital Saeedabad	Taluka Hospital	25.959014	68.375756
Matiari	Hala District Hospital	District Headquarter Hospital	25.818910	68.417196
Nasarpur*	RHC Nasarpur	RHC	25.515687	68.621544
Oderolal Station	RHC Oderolal Station	RHC	25.636002	68.595958
Sekhat	RHC Khyber	RHC	25.68128	68.511284
Matiari	Ali Medical Center & Maternity Home	MHC	25.59605	68.445869
Matiari	Noor Jahan Clinic & Maternity Home	MHC	25.596633	68.444591
Matiari	Haq Bahod Clinic & Maternity Home	MHC	25.501785	68.464783

Union council is the smallest administrative unit in Pakistan.

*Nasarpur is not located in Matiari district but is in close proximity of the union council.

BHU = Basic Health Unit, RHC = Rural Health Center, MHC = Maternal Health Center.
